# Use of cross-border healthcare services among ethnic Danes, Turkish immigrants and Turkish descendants in Denmark: a combined survey and registry study

**DOI:** 10.1186/1472-6963-12-390

**Published:** 2012-11-13

**Authors:** Signe Smith Nielsen, Suzan Yazici, Signe Gronwald Petersen, Anne Leonora Blaakilde, Allan Krasnik

**Affiliations:** 1 University of Copenhagen, Department of Public Health, Section for Health Services Research, Center for Healthy Aging and Danish Research Centre for Migration, Ethnicity and Health, Øster Farimagsgade 5A, DK-1014, Copenhagen, Denmark; 2 Akdeniz University, Department of Gerontology; Dumlupınar Bulvarı, 07059, Antalya, Turkey; 3 University of Copenhagen, Center for Healthy Aging, Department of Ethnology, Njalsgade 80, DK-2300, Copenhagen, Denmark

**Keywords:** Healthcare utilization, Medicine, Cross-border, Medical tourism, Migrant, Descendant, Continuity of care, Turkey, Denmark, Patient safety

## Abstract

**Background:**

Healthcare obtained abroad may conflict with care received in the country of residence. A special concern for immigrants has been raised as they may have stronger links to healthcare services abroad. Our objective was to investigate use of healthcare in a foreign country in Turkish immigrants, their descendants, and ethnic Danes.

**Methods:**

The study was based on a nationwide survey in 2007 with 372 Turkish immigrants, 496 descendants, and 1,131 ethnic Danes aged 18–66. Data were linked to registry data on socioeconomic factors. Using logistic regression models**,** use of doctor, specialist doctor, hospital, dentist in a foreign country as well as medicine from abroad were estimated. Analyses were adjusted for socioeconomic factors and health symptoms.

**Results:**

Overall, 26.6% among Turkish immigrants made use of cross-border healthcare, followed by 19.4% among their descendants to 6.7% among ethnic Danes. Using logistic regression models with ethnic Danes as the reference group, Turkish immigrants were seen to have made increased use of general practitioners, specialist doctors, hospitals, and dentists in a foreign country (odds ratio (OR), 5.20-6.74), while Turkish descendants had made increased use of specialist doctors (OR, 4.97) and borderline statistically significant increased use of hospital (OR, 2.48) and dentist (OR, 2.17) but not general practitioners. For medicine, we found no differences among the men, but women with an immigrant background made considerably greater use, compared with ethnic Danish women. Socioeconomic position and health symptoms had a fairly explanatory effect on the use in the different groups.

**Conclusions:**

Use of cross-border healthcare may have consequences for the continuity of care, including conflicts in the medical treatment, for the patient. Nonetheless, it may be aligned with the patient’s preferences and thereby beneficial for the patient. We need more information about reasons for obtaining cross-border healthcare among immigrants residing in European countries, and the consequences for the patient and the healthcare systems, including the quality of care. The Danish healthcare system needs to be aware of the significant healthcare consumption by immigrants, especially medicine among women, outside Denmark’s borders.

## Background

The global market and increasing global mobility introduce new dimensions of healthcare, in the form of the mobile patient, creating new possibilities and challenges for patients, providers, and the healthcare system. Patient mobility is defined as the ‘deliberate movements across international borders of patients seeking planned health care’ [1] and takes a variety of forms, ranging from international healthcare service agreements, people living in border areas, temporary visitors abroad, expatriates and immigrants seeking healthcare in their country of origin, to medical tourism, when wealthier patients seek the best care available or when lower socio-economic groups borrowing money or using savings to travel out of country for lower costs treatments than they may have to pay for private services in own country [2]. Five motives to seek healthcare in a foreign country have been identified: familiarity, availability, cost, quality, and bioethical legislation [3].

A cross-European survey showed that 4% of European Union citizens received treatment in another EU member state, ranging from 2% of the population in Sweden, to 6% in Denmark, and 20% in Luxembourg [4]. Although cross-border patient mobility still comprises a relatively small proportion of all elective care [5], it raises a concern regarding patient safety and continuity of care [5-7], which is an essential element of quality treatment and outcome. Special concern for the continuity of care and interference of medicine from abroad has been raised with regard to immigrants, as they may have stronger links to healthcare services abroad/in their home countries.

Denmark is a “new” immigration country; and the use of cross-border healthcare among immigrants is also a new phenomenon on the healthcare agenda. Due to the economic boom in the 1960s and early 1970s, the Danish state needed unskilled labour and therefore invited workers from primarily Turkey and Pakistan to Denmark. In 1973, Denmark stopped labour immigration, yet the immigrants who already had work and residence permits were allowed to stay. In the following years, the previous labour immigration flow was superseded by refugees and reunited family members [8]. Today, Turkish immigrants and their descendants constitute the largest immigrant group in Denmark, comprising 1.1% of the total population, equivalent to 60,390 persons [9]. Overall, immigrants and their descendants constitute 10.4% of the Danish population, of whom two thirds are non-Western immigrants [9]. Non-Western immigrants, including Turkish immigrants, are more often unemployed, social welfare recipients [10], and have lower incomes and educational qualifications compared with ethnic Danes [11]. The Danish healthcare system is characterised by a universal welfare model, whereby universal coverage is provided to all persons with residence permits, funded mainly via taxation. Access to the healthcare system is free, except for services such as dentistry, as well as prescription medicine requiring co-payment [12]. Until 2008, the Danish national health insurance covered all expenses to medical attention and treatment as well as prescription medicine obtained during holidays and study visits of up to one month in European countries and countries around the Mediterranean Sea, including Turkey, for persons with permanent residence in Denmark. Dentistry and, in principle, expenses for the treatment of chronic diseases or other diseases diagnosed before the time of departure were not covered. After 2008, only healthcare obtained in EU countries, Faroe Island, Greenland, Switzerland, Norway and a number of small European states (e.g. Lichtenstein) are covered.

Only very few studies have investigated immigrants’ use of healthcare abroad. In a US study, it was estimated that 952,000 Californian adults used healthcare in Mexico, of whom 488,000 were Mexican immigrants. Mexican immigrants in the US who had been the US for a long time had the highest rate of healthcare use in Mexico, at 15%, and US-born non-Latino whites the lowest rate (2.1%) [13]. An Irish qualitative study showed that patient mobility was most evident in the case of elective healthcare, and that immigrants were most likely to access healthcare in their host countries at times of emergency or crisis [2].

Cross-border healthcare use raises the concerns of continuity of care and interference of medicine from abroad, which may be more evident in immigrant populations, due to their familiarity with and potential preference for healthcare services in their countries of origin. So far, a review has pointed at major gaps in the evidence base for use of cross-border healthcare, including the patient profile of those seeking care abroad [6], and researchers have highlighted that there is an imperative for more empirical data on use of cross-border healthcare to be collected, in particular on the size of the use [7]. To our knowledge, the use of cross-border healthcare by Turkish immigrants has hitherto not been addressed in scientific literature, and no European studies have quantified immigrants’ cross-border healthcare use. Our objective was to investigate and compare cross-border use of healthcare among Turkish immigrants living in Denmark, their descendants, and ethnic Danes.

## Methods

### Study design and study population

In 2007, we conducted a nationwide survey of health and health behaviour among persons residing in Denmark aged 18–66 years who were: 1) ethnic Danes; 2) immigrants from the seven largest non-Western immigrant groups residing in Denmark for a minimum of three years; and 3) descendants of the two immigrant groups with the longest duration of stay in Denmark. According to Statistics Denmark, *ethnic Danes* are defined as persons with at least one parent born in Denmark with Danish citizenship; *immigrants* are defined as persons who were born in a foreign country to parents without Danish citizenship; and *descendants* are defined as persons born in Denmark with parents who were neither Danish citizens nor born in Denmark.

Under Danish law, only investigations which include human biological material must be reported to and approved by the Danish National Committee on Biomedical Research Ethics; this does not apply to questionnaire surveys, interview studies, and registry research surveys. This study required permission from the Danish Data Protection Agency only [14], and this permission was granted.

The selection procedure for participation in the survey was conducted in close collaboration with Statistics Denmark. Based on the assigned Civil Personal Registration Number (CPR number), Statistics Denmark randomly drew an equal number of persons from each immigrant and descendant group (an average of 1,072 per group), as well as 1,800 ethnic Danes, totalling a gross sample of 11,450 persons selected to participate in the survey. The CPR number contains information on sociodemographic characteristics, including country of birth and citizenship, and allows linkage of information over time to all other Danish registries. Persons who had died, emigrated, or were subject to research protection (N=1,935), were excluded. The net sample consisted of 9,515 persons: 4,952 responded, representing a response rate of 52.1%.

For this study, we restricted the sample to ethnic Danes, as well as Turkish immigrants and Turkish descendants, who constitute the largest immigrant and descendant group in Denmark. The final sample consisted of 372 Turkish immigrants, 496 descendants, and 1,131 ethnic Danes. The response rate was higher among ethnic Danes (73.3%) than among Turkish immigrants (48.3%) and Turkish descendants (55.4%).

### Survey data collection

A survey that includes different immigrant groups presents challenges in terms of the recruitment of participants, the validity of survey instruments, the study design, ensuring a high response rate, and the interpretation and communication of results. To accommodate these challenges, we carried out a pre-study by conducting qualitative interviews of 1–2 hours’ duration with a representative from each of the immigrant groups, as a starting point. The interviews formed the basis for the choice of study design and data collection (telephone interviews), as well as the decision to conduct a campaign before the start of the interviews in order to inform the immigrant groups about the upcoming survey.

To ensure cultural appropriateness, the questionnaire was prepared in close collaboration with representatives from each immigrant group, interpreters, and students with an immigrant background. Finally, the questionnaire was translated into the mother tongues of the immigrant groups using a forward/backward translation procedure.

To inform the potential respondents about the study and the impending telephone interview, all persons in the net sample were sent a letter in Danish and in the person's mother tongue. The sender of the letter was the “Partnership” formed by the Danish National Board of Health, three Danish regions, the three largest Danish municipalities, the National Institute of Public Health as well as the University of Copenhagen. In June-August 2007, computer-assisted telephone interviews were conducted by bilingual interviewers. In addition, participation via the Internet was offered.

### Variables

The exposure variable was country of origin, categorised as ethnic Danes, Turkish immigrants, and Turkish descendants. Concerning use of healthcare services in a foreign country, which were the outcome variables, participants were asked: “Within the last year, have you turned to one of the following healthcare services [general practitioner (GP), dentist, specialist doctor, hospital] in another country than Denmark?”; and “Within the last year, have you received medication from abroad?” The answer categories were yes and no. Survey data was linked to registry data from the Danish Demographic Database and the Integrated Database for Labour Market Research for the covariates on sociodemographic and socioeconomic information in 2007 (age, sex, marital status, education, employment, household income (the latter was adjusted for number of adults in the household)). Finally, the covariate health status was based on survey data, using a slightly modified SF-12 [15], with a sum of physical symptoms and mental symptoms. The two sum scores consisted of 6 items each, where the best possible answer was scored 0 and the worst 4. Variables were categorised as in Table 1, except health status, which was used as a continuous variable.

**Table 1:** Distribution of sociodemographic and socioeconomic characteristics, utilization of healthcare services in a foreign country and self-perceived health by country of origin

	**Denmark**	**Turkey**	**Descendant Turkey**
	**N (%)**	**N (%)**	**N ( %)**
**Response rate**	(73.3)	(48.3)	(55.4)
**Totals**	1,131 (100)	372 (100)	496 (100)
**SOCIODEMOGRAPHIC FACTORS**			
**Age**			
18-29	195 (17)	65 (18)	449 (91)
30-39	228 (20)	144 (39)	47 (9)
40-49	287 (25)	105 (28)	-
50-66	421 (37)	58 (16)	-
**Sex**			
Male	553 (49)	189 (51)	270 (54)
Female	578 (51)	183 (49)	226 (46)
**Marital Status**			
Married or living w/partner	634 (56)	311 (84)	127 (26)
Not married/divorced/widow	493 (44)	61 (16)	367 (74)
**Education**			
Unknown	8 (1)	66 (18)	9 (2)
Primary	265 (24)	203 (55)	358 (73)
Secondary	524 (47)	82 (22)	105 (21)
Tertiary	330 (29)	21 (6)	22 (5)
**Employment**			
Employed	944 (88)	213 (65)	301 (63)
Unemployed	25 (2)	47 (14)	25 (5)
Outside labour market	109 (10)	68 (21)	155 (32)
**Household income (**DKK)			
<99,999	64 (6)	52 (15)	166 (40)
100,000-199,999	192 (18)	169 (47)	145 (35)
200,000-299,999	371 (34)	112 (31)	82 (20)
>300,000	454 (42)	25 (7)	20 (5)
**HEALTH STATUS**			
Physical Health Symptoms*			
0-5	922 (82)	216 (58)	420 (85)
6-10	115 (10)	67 (18)	49 (10)
11-15	50 (4)	33 (9)	20 (4)
16-20	29 (3)	32 (9)	4 (1)
21≤ (26)	15 (1)	24 (7)	3 (1)
Mean	3.42	6.50	2.82
Mental Health Symptoms*			
0-5	853 (75)	199 (54)	314 (63)
6-10	213 (19)	112 (30)	149 (30)
11-15	41 (4)	28 (8)	23 (5)
16-20	18 (2)	25 (7)	9(2)
21≤ (30)	6 (1)	8 (2)	1 (0)
Mean	4.36	6.48	5.05
**HEALTHCARE UTILIZATION IN A FOREIGN COUNTRY**			
**Have used healthcare in a foreign country**	76 (6.7)	99 (26.6)	96 (19.4)
**General practitioner**	49 (4.3)	56 (15.1)	48 (9.7)
**Specialist doctor**	18 (1.6)	46 (12.4)	21 (4.2)
**Hospital**	16 (1.4)	35 (9.4)	29 (5.8)
**Dentist**	35 (3.1)	34 (9.1)	29 (5.8)
**Medicine**	25 (2.2)	54 (14.5)	44 (8.9)

* 0 representing the best possible health.

### Statistical analysis

Chi^2^-tests were used to compare unadjusted proportions of the different outcome variables and covariates by country of origin. We used binary logistic regression to examine the association of country of origin with each dependent variable. Sociodemographic and socioeconomic factors, as well as health status, were included stepwise (one a time and several a time) in the models, as control and intermediate variables. We also investigated interactions between country of origin and sex. Analyses were conducted using SPSS PASW Statistics version 19.

## Results

The distribution of the variables included by ethnic Danes, Turkish immigrants, and Turkish descendants is presented in Table 1. Turkish immigrants and their descendants were more likely to be married/have a partner, have primary education, have a low income, be unemployed, and be outside the workforce than ethnic Danes (P<.001). Furthermore, Turkish immigrants were also more likely to be younger than ethnic Danes (P<.001). Compared with ethnic Danes, Turkish immigrants reported more health symptoms, whereas descendants reported fewer physical health symptoms, but more mental health symptoms (P≤.001-.009). In unadjusted analyses, compared with ethnic Danes, Turkish immigrants and their descendants made greater use of GPs, specialist doctors, hospitals, dentists, and medicine in a foreign country (P<.001). Overall, Turkish immigrants made the highest use of cross-border healthcare (a total of 26.6%), ranging from 9.1% (dentists) to 15.1% (GPs), followed by Turkish descendants (a total of 19.4%), ranging from 4.2% (specialist doctors) to 9.7% (GPs), while ethnic Danes made the lowest use (a total of 6.7%), ranging from 4.3% (GPs) to 1.4% (hospitals).

### Use of general practitioners, specialist doctors, hospitals and dentists

After adjustment for sex and age, Turkish immigrants made increased use of all healthcare services in a foreign country (GPs, specialist doctors, hospitals, and dentists) (odds ratio (OR), 3.13-10.17, Table 2, Model II) compared with ethnic Danes. The largest differences between Turkish immigrants and ethnic Danes were seen for specialist doctors (OR, 10.17), hospitals (OR, 8.28) and dentists (OR, 3.13). Further adjusted for marital status and socioeconomic factors, the differences between Turkish immigrants and ethnic Danes varied somewhat; for use of GPs and dentists, the difference increased, and for use of specialist doctors and hospitals, the difference decreased (Model IV). On adjusting further for physical and mental health status, the difference decreased for specialist doctors and hospitals and remained somewhat similar for GPs and dentists (Model V). Nevertheless, for use of all healthcare services, after adjustment of all variables included, we observed higher use among Turkish immigrants compared with ethnic Danes (OR, 4.49-6.74 dependent on healthcare service, Model V).

**Table 2:** Adjusted Odds Ratios (95% CI) for healthcare utilization in a foreign country in 2007 by country of origin

**Country of Origin**	**Doctor**									
	**Model I**		**Model II**		**Model III**		**Model IV**		**Modul V**	
	**OR (95% CI)**	***P***	**OR (95% CI)**	***P***	**OR (95% CI)**	***P***	**OR (95% CI)**	***P***	**OR (95% CI)**	***P***
**General practitioner**										
Denmark	1.00		1.00		1.00		1.00		1.00	
Turkey	**3.91 (2.61-5.86)**	<.000	**3.86 (2.54-5.87)**	<.000	**4.56 (2.91-7.13)**	<.000	**4.25 (2.38-7.60)**	<.000	**4.49 (1.92-6.32)**	<.000
Turkey Descendants	**2.37 (1.57-3.58)**	<.000	**1.72 (1.02-2.90)**	.041	**1.91 (1.13-3.26)**	.017	1.30 (0.69-2.42)	.416	1.19 (0.64-2.23)	.579
**Specialist doctor**										
Denmark	1.00		1.00		1.00		1.00		1.00	
Turkey	**8.73 (4.99-15.26)**	<.000	**10.17 (5.65-18.30)**	<.000	**12.29 (6.53-23.14)**	<.000	**9.31 (4.02-21.58)**	<.000	**6.74 (2.84-16.01)**	<.000
Turkey Descendants	**2.73 (1.44-5.18)**	.002	**6.10 (2.51-14.19)**	<.000	**7.31 (2.91-18.36)**	<.000	**5.92 (2.06-16.96)**	.001	**4.97 (1.72-14.41)**	.003
**Hospital**										
Denmark	1.00		1.00		1.00		1.00		1.00	
Turkey	**7.24 (3.96-13.24)**	<.000	**8.28 (4.39-15.62)**	<.000	**9.22 (4.71-18.01)**	<.000	**7.95 (3.33-18.99)**	<.000	**5.70 (2.33-13.93)**	<.000
Turkey Descendants	**4.33 (2.33-8.04)**	<.000	**4.07 (1.84-9.03)**	.001	**4.41 (1.95-9.94)**	<.000	**3.12 (1.19-8.18)**	.021	2.48 (0.95-6.48)	.063
**Dentist**										
Denmark	1.00		1.00		1.00		1.00		1.00	
Turkey	**3,15 (1.94-5.13)**	<.000	**3.13 (1.89-5.18)**	<.000	**3.34 (1.96-5.68)**	<.000	**5.72 (2.85-11.45)**	<.000	**5.20 (2.57-10.54)**	<.000
Turkey Descendants	**1.95 (1.18-3.22)**	.010	1.58 (0.82-3.02)	.170	1.72 (0.89-3.33)	.109	**2.28 (1.04-4.97)**	.039	2.17 (0.996-4.74)	.051

95% CI indicates 95% confidence interval.

Statistically significant variables (P <.05) are depicted in bold type.

Model I: Using binary logistic regression to adjust for country of origin.

Model II: Using binary logistic regression to adjust for country of origin, sex, age.

Model III: Using binary logistic regression to adjust for country of origin, sex, age, and marital status.

Model IV: Using binary logistic regression to adjust for country of origin, sex, age, marital status, education, employment status, and household income.

Model V: Using binary logistic regression to adjust for country of origin, sex, age, marital status, education, employment status, household income, physical health symptoms, and mental health symptoms.

All estimates are adjusted for all covariates, even though some of the covariates are statistically non-significant.

Turkish descendants made generally lower use of healthcare services in a foreign country compared with their parental generation, but higher use compared with ethnic Danes (Model I). After adjusting for sex and age, Turkish descendants made increased use of all healthcare services in a foreign country (OR, 1.72-6.10 dependent on healthcare service, Model II), except dentists, compared with ethnic Danes. Adjustment for marital status and socioeconomic factors further narrowed the differences, becoming statistically insignificant for use of GPs, but statistically significant for use of dentists (Model IV). On adjusting further for health symptoms, we observed an increased use of specialist doctors (OR, 4.97), and a similar use of GPs in a foreign country compared to ethnic Danes (Model V). Furthermore, there were tendencies of increased use of hospitals and dentists among Turkish descendants compared with ethnic Danes as the numbers became just borderline statistically insignificant at alpha 0.05 (Model V). The interaction of country of origin by sex was not found to be statistically significant.

### Use of medicine

For medicine received abroad, the interaction of country of origin by sex was found to be statistically significant (P=0.019) (Table 3). After adjusting for age, we observed higher use of medicine from abroad among immigrant Turkish men (OR, 2.59), but not among their descendant counterparts, compared with ethnic Danish men; while we observed considerably higher use of medicine from abroad among immigrant Turkish women (OR, 15.57) and their descendants (OR, 6.87), compared with ethnic Danish women (Model II). On also adjusting for marital status and socioeconomic status, we found no statistically significant difference between the groups of men, while the differences between the groups of women increased (Model IV). On adjusting further for physical and mental health symptoms, we found no differences in the use of medicine from abroad among ethnic Danish men, immigrant Turkish men, and their descendant counterparts, but we found considerably higher use among immigrant Turkish women (OR, 14.00) and their descendant counterparts (OR, 4.08) compared with ethnic Danish women (Model V).

**Table 3:** Adjusted Odds Ratios (95% CI) for medicine received from abroad in 2007 by country of origin and sex

**Men**										
**Country of Origin**	**Model I**		**Model II**		**Model III**		**Model IV**		**Modul V**	
	**OR (95% CI)**	***P***	**OR (95% CI)**	***P***	**OR (95% CI)**	***P***	**OR (95% CI)**	***P***	**OR (95% CI)**	***P***
**Men**										
Denmark	1.00		1.00		1.00		1.00		1.00	
Turkey	**3.07 (1.40-6.74)**	.005	**2.59 (1.15-5.84)**	.021	**2.61 (1.10-6.17)**	.029	1.89 (0.57-6.25)	.295	1.65 (0.48-5.72)	.430
Turkey Descendants	**2.62 (1.24-5.52)**	.012	1.61 (0.63-4.10)	.316	1.61 (0.63-4.12)	.319	2.14 (0.70-6.51)	.181	1.82 (0.58-5.73)	.309
**Women**										
Denmark	1.00		1.00		1.00		1.00		1.00	
Turkey	**13.62 (6.98-25.60)**	<.000	**15.57 (7.73-31.37)**	<.000	**18.87 (8.94-39.83)**	<.000	**18.78 (7.2-48.91)**	<.000	**14.00 (5.29-37.06)**	<.000
Turkey Descendants	**6.67 (3.33-13.37)**	<.000	**6.87 (2.83-16.64)**	<.000	**7.81 (3.14-19.44)**	<.000	**4.93 (1.67-14.56)**	.004	**4.08 (1.39-11.96)**	.010

95% CI indicates 95% confidence interval.

Statistically significant variables (P <.05) are depicted in bold type.

Model I: Using binary logistic regression to adjust for country of origin.

Model II: Using binary logistic regression to adjust for country of origin, age.

Model III: Using binary logistic regression to adjust for country of origin, age, and marital status.

Model IV: Using binary logistic regression to adjust for country of origin, age, marital status, education, employment status, and household income.

Model V: Using binary logistic regression to adjust for country of origin, age, marital status, education, employment status, household income, physical health symptoms, and mental health symptoms.

All estimates are adjusted for all covariates, even though some of the covariates are statistically non-significant.

## Discussion

In this combined survey and registry-based study, we observed higher use of all somatic healthcare services (GPs, specialist doctors, hospitals, and dentists) in a foreign country among Turkish immigrants compared with ethnic Danes, also after adjustment for sociodemographic and socioeconomic factors, as well as health status. Turkish descendants made increased use of specialist doctors and there were tendencies of increased use of hospitals and dentists, while we found no statistically significant differences in use of GPs in a foreign country compared with ethnic Danes, after adjustment for sociodemographic and socioeconomic factors, as well as health status. For medicine received from abroad, we found no statistically significant differences among the men, but immigrant Turkish women and their descendant counterparts made extensively higher use compared with ethnic Danish women. Socioeconomic factors and health status could explain some, but not the full effect on the use made by the different groups.

### Strengths and limitations

The strengths of the study include interviews of the population in their preferred languages and the use of survey data linked to high-quality registry data. Additionally, we examined the use of different healthcare providers abroad, taking into account different population groups’ preferences for types of medical care. Several limitations also apply. Firstly, the survey questions on the use of healthcare in a foreign country give insufficient information: we do not have information on the country(ies) from which the healthcare services are obtained, nor do we have information on which types of healthcare (e.g. which types of medicine) are obtained. Different definitions of healthcare or medication between the groups included may also be at stake. Secondly, the low participation rate of the Turkish immigrant and descendant groups may lead to underestimation of the use of healthcare abroad, as non-respondents are likely to have low socioeconomic status and may have more problems with contacts with Danish authorities, including the healthcare system. Nevertheless, it could also lead to overestimation if non-respondents use cross-border healthcare services less due to their poor financial situation. Thirdly, only immigrants who had resided in Denmark for at least three years were included in the study. Information on newly arrived immigrants’ use of healthcare may also be of interest. Fourthly, use of self-reported health symptoms as a measure of health is problematic, leading to potential residual confounding. Cultural differences in the health of one’s reference group, different expectations of one’s health, and differences in response style, or in the connotation of the symptom questions, may play a role, so that it is uncertain whether the symptom scales function equivalently in all three groups. Fifthly, the registry information lacked accuracy in two aspects: a) information on education was obtained from a questionnaire sent annually to all immigrants. Based on this information, Statistics Denmark classifies immigrants’ education according to Danish standards, but this is subject to uncertainty and lack of information; b) unemployment was defined as full unemployment in week 48, which is a weak indicator and does not take the length of unemployment into account.

### Reasons for seeking cross-border healthcare

Reasons for seeking healthcare services across borders/in the country of origin by migrants are complex and deeply rooted in the specific cultural background of the migrants, as well as formal and informal access to the healthcare system in the host country. An Irish qualitative study on recently arrived immigrants showed that the immigrants were confused about the Irish healthcare system, including their entitlements, procedures for obtaining them, and the services available. The immigrants sought to fill some, and occasionally most, of their healthcare needs in their home country, e.g. during summer holidays; and not only for specialised procedures, but also for non-specialised routine check-ups and minor treatments [2]. Other studies indicate other rationales for seeking healthcare services in their country of origin. Korean immigrants in New Zealand, who represent a relatively young and skilled immigrant group, sought effective care, as well as better and faster treatment, in culturally comfortable settings in Korea [16]. Treatment in Korea was considered to be faster and of higher quality by the Korean immigrants. Combined with feeling comfortable and safe, and overcoming cultural and linguistic barriers, this seemed to outweigh travel expenses [16]. Mexicans living far from the border in the US sought medical treatment in Mexico due to unsuccessful treatment in the US, lack of (financial) access to care in the US, and a preference for Mexican care [17]. For Californian Mexicans (living relatively closer to the border), the predictors of cross-border healthcare use by immigrants were found to be: need, lack of health insurance, delay in the seeking of care, more recent immigration and limited English language proficiency [13].

The case of Turkish immigrants seeking healthcare services in Turkey is somewhat similar to the Korean immigrants in New Zealand seeking healthcare in Korea, since the healthcare systems in the two host countries and in the countries of origin, respectively, to a large extent share similar characteristics. New Zealand and Denmark have a GP gatekeeper system, whereas in Korea and Turkey patients go directly to specialists in hospitals [12,16,18]. However, while the Korean immigrants in New Zealand had to pay out-of-pocket for their treatment in Korea, the Turkish immigrants in Denmark had free-of-charge or partly reimbursed medical treatment, including prescription medicine, in Turkey, as this was covered by the Danish national health insurance at the time of the data collection (until 2008). Yet, for additional services such as dentistry, or in the case of chronic diseases, the Turkish immigrants residing in Denmark had to pay for the services in Turkey themselves. There might also be differences in the accessibility of specialist and hospital care between the healthcare system in Denmark and in Turkey. With reference to the Korean immigrants, it is likely that Turkish immigrants would be more likely to use private services in Turkey, and accordingly receive fast and efficient services in a culturally familiar setting with healthcare providers one trust and thereby avoid waiting lists in Denmark. Future research may focus on how and why and which types of cross-border healthcare have been obtained, as this could be useful to improve measures and to make recommendations for the European healthcare systems.

### Alternative or supplementary healthcare?

A recent Danish study showed that Turkish immigrants and their descendants made considerably higher use of somatic healthcare services (GPs, specialists in private practice, emergency rooms, hospitals) but less use of dentists in Denmark, compared with ethnic Danes—also after taking sociodemographic and socioeconomic factors and health status into account [11]. The present study shows that these utilization differences in the Danish healthcare system cannot be explained by differences in the use of cross-border healthcare by Turkish immigrants, their descendants, and ethnic Danes. One exception is the use of dentists by immigrants, and to some extent by descendants. Since this study showed higher use of dentists in a foreign country among Turkish immigrants and borderline higher use among their descendants compared with ethnic Danes, the lower use of dentists by immigrants and their descendants in Denmark may be of less concern. Nonetheless, less than 10% of Turkish immigrants and less than 6% of Turkish descendants consulted a dentist in a foreign country (Table 1). From these two studies, it seems that use of cross-border healthcare among Turkish immigrants residing in Denmark should be considered to be supplementary healthcare, and not as an alternative.

### Informal barriers to the host country healthcare system

The observations of different consumption patterns of use of healthcare abroad may be due to informal barriers to the Danish healthcare system, including inadequate doctor-patient communication, provider insecurity, different healthcare-seeking behaviour, and patient preferences and expectations. As Danish studies have shown dissatisfaction with the doctor-patient encounter from healthcare professionals and from non-Western immigrants [19,20], this is likely to lead to the increased use of healthcare services in the immigrants’ home countries. Furthermore, immigrants may have longer stays abroad/in their home countries, which naturally add to increased cross-border healthcare consumption. However, the Irish study of recently arrived immigrants found that the main reasons for seeking healthcare in their home countries appeared to be issues of affordability and perception of the quality of care [2]. Unlike the common idea that seeking healthcare in a more familiar context, such as their country of origin, may in the case of migrants be due to (perceived) social, cultural, religious, and linguistic differences, as was found by the Korean immigrants in New Zealand [1,16], the Irish study suggested that this was due to insufficient information about the Irish health system. Even though the immigrants did not report cultural and linguistic difficulties, the types of complaints, including quality of services, made the authors consider that they might be related to feelings of alienation [2]. Our study differs significantly from the Irish study in that the current study population has resided in Denmark for at least three years, and in that the descendants were born in Denmark. Consequently, complete unfamiliarity with the Danish healthcare system is not likely to be at stake, yet cultural barriers, including communication and affordability, especially in the case of dentists and medicine, are likely to be present.

### Generational differences in the use of healthcare

Only few studies have looked into generational differences in the use of healthcare. Utilisation patterns for immigrants’ descendants may be of particular interest for prioritising in the healthcare system, as we may assume that they converge into the pattern for ethnic Danes. This study showed that, compared with ethnic Danes, Turkish descendants made increased use of specialist doctors and borderline increased use of hospitals and dentists, and female descendants made increased use of medicine from abroad. For GP services, we found no statistically significant difference between Turkish descendants and ethnic Danes. One explanation could be that, in important cases, a second opinion from a specialist/hospital in the country of origin is still associated with a feeling of security and quality, while the use of dentist in a foreign country may be due to lower prices. The use of prescription medicine from a foreign country may be due to affordability, the availability of over-the-counter medicine in Turkey that would be prescription medicine in Denmark, as well as the unavailability of specific medical drugs used in Turkey on the Danish market. The descendants of Turkish immigrants in Denmark are likely to be familiar with the Turkish healthcare system, due to regular visits to Turkey and their parents’ use of services in Turkey; so that the descendants know both the Danish and the Turkish healthcare systems and have the skills to navigate in them and between them, in order to get the best and fastest treatment.

### Implications for healthcare in countries of residence

Use of cross-border healthcare services may impose additional strains on the healthcare systems in countries of residence, as it may disrupt continuity of care for patients, including giving patients mixed information and diverging treatment, and may create professional obstacles for healthcare providers. Another implication for healthcare in countries of residence is patient safety regarding contact with resistant bacteria or the increase of antibiotic resistance by using antibiotic too easy. Furthermore, healthcare obtained in a foreign country may entail that the patient does not achieve relevant rehabilitation, etc. in the home country, with the risk of poorer health outcomes and even more fragmented care [5]. For chronic diseases that to a larger extent require continuity of care, cross-border healthcare use may be a complicating factor. However, the use of healthcare abroad can also be looked upon as a supplement and may thus be beneficial for the patient. Questions about the quality of services abroad have been raised, but relatively little is known about readmission, morbidity, and mortality subsequent to medical treatment abroad [6]. However, intake of medicine from foreign countries may be highly problematic, as in many cases the quality is questionable due to the lack of proper control or the adulteration of such medicine, making it dangerous for the patient [21].

Patient mobility is increasing worldwide and is high on the agendas of national healthcare systems and health authorities [7,22]. Immigrants and their descendants have a background from (at least) two countries, and more affluent members of this group in particular may possess greater knowledge and capability of navigating different healthcare systems, making them better able to exploit services that are most beneficial to them. With increasing globalisation, more and more individuals will be in a similar situation.

## Conclusion

In conclusion, the increased use of cross-border healthcare services, especially among Turkish immigrants, may have consequences for continuity of care, including conflicts in medical treatment. In particular, the high uptake of medicines from abroad by Turkish female immigrants and their female descendants may be of special concern. However, use of cross-border healthcare may also be beneficial for patients since it may be aligned with patient preferences, and patients may receive faster treatment as well as be beneficial for the healthcare system in the host country since it may reduce waiting lists and reduce expenditures. We need more information about reasons for obtaining cross-border healthcare among immigrants residing in European countries, and the consequences for the patient and the healthcare systems, including the quality of care. The Danish healthcare system needs to be aware of the significant healthcare consumption by immigrants outside Denmark’s borders.

## Competing interests

The authors declare that they have no competing interests.

## Authors’ contributions

SSN conceived the study, participated in its design, was involved in the data collection, performed the statistical analyses and interpretation of data and drafted the article. SY participated in the interpretation of data and revised the article. SGP participated in the interpretation of data and revised the article. ALB participated in the interpretation of data and revised the article. AK conceived the study, was involved in the data collection, participated in the interpretation of data and revised the article. All authors read and approved the final manuscript.

## Pre-publication history

The pre-publication history for this paper can be accessed here:

http://www.biomedcentral.com/1472-6963/12/390/prepub
